# Time Domain Characterization of the Cole-Cole Dielectric Model

**DOI:** 10.2478/joeb-2020-0015

**Published:** 2020-12-31

**Authors:** Sverre Holm

**Affiliations:** 1 Department of Physics, University of Oslo, Oslo, Norway

**Keywords:** Cole-Cole, Curie-von Schweidler, Kohlrausch, Constant phase element

## Abstract

The Cole-Cole model for a dielectric is a generalization of the Debye relaxation model. The most familiar form is in the frequency domain and this manifests itself in a frequency dependent impedance. Dielectrics may also be characterized in the time domain by means of the current and charge responses to a voltage step, called response and relaxation functions respectively. For the Debye model they are both exponentials while in the Cole-Cole model they are expressed by a generalization of the exponential, the Mittag-Leffler function. Its asymptotes are just as interesting and correspond to the Curie-von Schweidler current response which is known from real-life capacitors and the Kohlrausch stretched exponential charge response.

## Introduction

The Cole Cole model [1] is a generalization of the Debye dielectric relaxation model which fits measurements in many applications including the bioimpedance field, [2, Sec. 9.2.7]. One interpretation is that it represents a distribution of relaxation processes, each described by the Debye model. Since the Debye model has a simple time domain interpretation and both the current and charge responses to a voltage step are exponential, the Cole-Cole responses can therefore be expressed as sums of exponential functions. In practice, however, this result is often too complex to lend itself to interpretation.

In recent years, there has been a development in understanding of the responses of the Cole-Cole model found in a direct way. These results depend on the Mittag-Leffler function, a generalization of the exponential which is named after Gösta Mittag-Leffler (1846–1927). This function is rightly called the “queen function of fractional calculus” [3] showing the close link between non-integer derivatives and the Cole-Cole model. The asymptotes of the Mittag-Leffler function are just as important as the function itself and is what will be emphasized here.

There are two well-established results for non-ideal dielectrics. The first is that for a long time it has been known that the current response to a step voltage for a practical non-ideal capacitor often follows the Curie-von Schweidler power law:

(1) $$I(t)\propto t^{-\alpha \prime },$$

where *α*′ is an order which is defined after (19). In [4] such responses are measured for many practical capacitors and the law is attributed to Curie in 1889 and von Schweidler in 1907. This is especially relevant for non-ideal dielectrics.

The second is an even older result which is due to Kohlrausch who found that the discharge of a capacitor with glass as a dielectric medium in a Leiden jar follows a stretched exponential. The charge is:

(2) $$Q(t)\propto \text{exp}\left[-{\left(t/\tau *\right)}^{\alpha }\right],$$

where τ* is a time constant and *α* is given after (19). In [5] this is traced back to 1854. The result was later rediscovered by Williams and Watts [6] and it is often called the Kohlrausch-Williams-Watt model.

The purpose of this paper is to increase awareness of the time domain properties of the Cole-Cole model by collecting and interpreting some results from in particular [5, 7, 8]. The paper starts with the Debye model in order to define the relevant current and charge responses, called the response function and the relaxation function respectively. It will also be shown that both the Curie-von Schweidler power law and the Kohlrausch-Williams-Watt stretched exponential response are approximations to those of the Cole-Cole model. Finally, it is also shown that just as the Debye model corresponds to an ordinary partial differential equation for the constitutive law between the displacement field and the electric field, the Cole-Cole model corresponds to a similar equation but with non-integer, i.e. fractional derivatives.

## Definitions

The constitutive relation between the displacement field, *D*, and the electric field, *E* is:

(3) $$D=\varepsilon_{0}\varepsilon_{r}E=\varepsilon_{0}E+\chi E=\varepsilon_{0}E+P,$$

where *P* is the polarization charge density, *ɛ*_0_ is free space permittivity, *ɛ_r_* is relative permittivity, and *ξ* is susceptibility. In the time domain, *D*(*t*), represents a charge density.

Frequency-dependency can be given either for the susceptibility [5, 9] or for the permittivity [2]. The relationship between the two is:

(4) $$\chi =\varepsilon_{0}(\varepsilon_{r}-1).$$

There are two reasons why we consider the permittivity here. First, in the bioimpedance field *ɛ_r_* ≫ 1 so there is little difference in practice and *D* ≈ *P*. Second and more important, it is *ɛ_r_* which is directly reflected in the properties of the macroscopic capacitance of the medium, and since impedance or capacitance is what can be measured, it makes sense to specify properties in terms of *ɛ_r_*. This is also how models are justified in the bioimpedance field as [2, Sec. 3.1.3] says: “Polarization P cannot itself be measured. Dielectric theory is therefore invariably linked with the concept of a capacitor formed by two plates with the dielectric in between.”

This capacitance of such a dielectric material is

(5) $$C=\frac{A}{d}\varepsilon_{0}\varepsilon_{r},$$

where *A* and *d* are the area and the plate distance of the capacitor. The capacitance is complex for the models considered here and its impedance is *Z* = (j*ωC*)^−1^. The frequency domain response to an input voltage is:

(6) $$I(\omega )=\frac{U(\omega )}{Z(\omega )}=\text{j}\omega \frac{\varepsilon_{0}A}{d}\varepsilon_{r}(\omega )U(\omega ).$$

When the input voltage is a step, *U*(*ω*) = (j*ω*)^−1^, this is:

(7) $$I_{\mathrm{step}}(\omega )=\frac{\varepsilon_{0}A}{d}\varepsilon_{r}(\omega ).$$

In the time domain, the current step response is found as the inverse Fourier transform of the relative permittivity.

The current charge relation is :

(8) $$I=A\cdot J=A\frac{\text{d}Q}{\text{d}t},$$

where *J*(*t*) is the charge density. Charge is therefore found by an integration of the result for the current plus a constant. Integration is equivalent to division by j*ω* in the frequency domain and therefore the charge response to a step in voltage is related to

(9) $$Q_{\mathrm{step}}(\omega )=\frac{I_{\mathrm{step}}(\omega )}{\text{j}\omega A}=\frac{\varepsilon_{0}}{d}\frac{\varepsilon_{r}(\omega )}{\text{j}\omega }.$$

## Debye model

As a reference and in order to establish terminology, the Debye model will first be analyzed for its current and charge responses. Its permittivity is

(10) $$\varepsilon_{r}(\omega )=\varepsilon_{\infty }+\frac{\varepsilon_{s}-\varepsilon_{\infty }}{1+\text{j}\omega \tau },$$

where *ɛ_s_* is the static value and *ɛ*_∞_ < *ɛ_s_* is the value at infinity frequency, *τ* is a characteristic time constant for the medium. The first term, represented by the constant *ɛ*_∞_, represents an ideal capacitor which is in parallel with a frequency-varying part.

### Time-domain characterization

The current response, (7), is

(11) $$\begin{array}{ll} I_{\mathrm{step}}(t) & =\frac{\varepsilon_{0}A}{d}{ℱ}^{-1}\left\{\varepsilon_{r}(\omega )\right\}\\ & =\frac{\varepsilon_{0}A}{d}\left(\varepsilon_{\infty }\delta (t)+\frac{\varepsilon_{s}-\varepsilon_{\infty }}{\tau }{\text{e}}^{-t/\tau }\right). \end{array}$$

The current has an initial impulse due to the charging of an ideal capacitor followed by a current that dies out with a time constant *τ*.

Likewise the charge response is given by (9) or by integration of the current:

(12) $$Q_{\mathrm{step}}(t)=\frac{1}{A}\int_{0}^{t}I_{\mathrm{step}}(u)\mathrm{du}.$$

This gives

(13) $$\begin{array}{ll} Q_{\mathrm{step}}(t) & =\frac{\varepsilon_{0}}{d}\left(\varepsilon_{\infty }-\left(\varepsilon_{s}-\varepsilon_{\infty }\right){\text{e}}^{-t/\tau }+K\right)\\ & =\frac{\varepsilon_{0}}{d}\left(\varepsilon_{s}-\left(\varepsilon_{s}-\varepsilon_{\infty }\right){\text{e}}^{-t/\tau }\right),t\geq 0, \end{array}$$

where *K* is a constant which is such that the initial value for the charge is proportional to *ɛ*_∞_. The charge therefore starts with this value and ends up to be proportional to *ɛ_s_* for large time. This is in agreement with the example in [2, Sec. 3.4.2].

### Characterization of general models

The models will be given in terms of a normalized permittivity which for the Debye model is:

(14) $$\varepsilon_{D}(\omega )=\frac{\varepsilon_{r}(\omega )-\varepsilon_{\infty }}{\varepsilon_{s}-\varepsilon_{\infty }}=\frac{1}{1+\text{j}\omega \tau }.$$

In order to characterize subsequent models, the two descriptions of [5] will be used. The first is the response function, *φ*(*t*), which characterizes the current response. It is given as the inverse Fourier transform of the normalized relative permittivity, *ɛ*(*ω*). In the Debye example this is

(15) $$\phi (t)={ℱ}^{-1}\left\{\varepsilon (\omega )\right\}={ℱ}^{-1}\left\{\frac{1}{1+\text{j}\omega \tau },\right\}=\frac{1}{\tau }{\text{e}}^{-t/\tau },$$

which can be recognized to be the main time-varying part of (11).

The second function is the relaxation function, *ψ*(*t*), which characterizes the charge. It is given by

(16) $$\psi (t)={ℱ}^{-1}\left\{\frac{1-\varepsilon (\omega )}{\text{j}\omega }\right\}={\text{e}}^{-t/\tau },\;t\geq 0,$$

which is seen in (13) with a negative sign. It is only for the simple Debye model that the two functions are the same.

The definitions of *φ*(*t*) and *ψ*(*t*) are such that they both are non-negative and non-increasing functions of time, meaning that the zero order derivative is positive and the first order derivative is negative. This pattern of sign changes repeats infinitely with the second order derivative positive and so on. This is what characterizes a completely monotone function and it ensures that it can be expressed as a continuous distribution of exponential functions and that the Laplace transform is non-negative [5]. In this way the physical realizability of the considered system is guaranteed [10].

### Constitutive law

The Debye model can also be expressed as a differential equation between D and E by combining (3) with a rearranged (10):

(17) $$(1+\text{j}\omega \tau )D(\omega )=\varepsilon_{0}\varepsilon_{s}E(\omega )+j\omega \tau \varepsilon_{0}\varepsilon_{\infty }E(\omega ).$$

Inverse Fourier transformation then gives:

(18) $$D(t)+\tau \frac{\text{d}D(t)}{\text{d}t}=\varepsilon_{0}\varepsilon_{s}E(t)+\tau \varepsilon_{0}\varepsilon_{\infty }\frac{\text{d}E(t)}{\text{d}t}.$$

This is the electrical equivalent of the standard linear solid or Zener model in linear viscoelasticity [7, 11].

## Cole-Cole model

The permittivity of the Cole-Cole model follows a more general power-law than the Debye model:

(19) $$\varepsilon_{r}(\omega )=\varepsilon_{\infty }+\frac{\varepsilon_{s}-\varepsilon_{\infty }}{1+{\left(\text{j}\omega \tau \right)}^{1-\alpha \prime }}=\varepsilon_{\infty }+\frac{\varepsilon_{s}-\varepsilon_{\infty }}{1+{\left(\text{j}\omega \tau \right)}^{\alpha }}.$$

In electromagnetics, the model is sometimes presented with an exponent of 1 − *α*′, so that *α*′ = 0 corresponds to the Debye model. It may also be expressed with an exponent *α* = 1 − *α*′ where 0 < *α* ≤ 1 and here that convention will be followed in order to conform to [5].

The permittivity of the Cole-Cole model in normalized form is:

(20) $$\varepsilon_{\mathrm{CC}}(\omega )=\frac{\varepsilon_{r}(\omega )-\varepsilon_{\infty }}{\varepsilon_{s}-\varepsilon_{\infty }}=\frac{1}{1+{\left(\text{j}\omega \tau \right)}^{\alpha }}.$$

### Time domain characterization

Section 3.1 of [5] gives the functions for the Cole-Cole model. The response function that characterizes the current response is:

(21) $$\phi_{\mathrm{CC}}(t)={ℱ}^{-1}\left\{\varepsilon_{\mathrm{CC}}(\omega )\right\}=\frac{1}{\tau }{\left(t/\tau \right)}^{\alpha -1}{\text{E}}_{\alpha ,\alpha }\left(-{\left(t/\tau \right)}^{\alpha }\right),$$

and the relaxation function which describes the charge is:

(22) $$\psi_{\mathrm{CC}}(t)={ℱ}^{-1}\left\{\frac{1}{\text{j}\omega }-\frac{\varepsilon_{\mathrm{CC}}(\omega )}{\text{j}\omega }\right\}={\text{E}}_{\alpha }\left(-{\left(t/\tau \right)}^{\alpha }\right).$$

The function E_*α*,*β*_ is the Mittag-Leffler function which is a generalization of the exponential function. The two-parameter Mittag-Leffler function is defined by

(23) $${\text{E}}_{\alpha ,\beta }(t)=\sum_{n=0}^{\infty }\frac{t^{n}}{\lceil \left(\alpha n+\beta \right)},\;0\;<\;\alpha \;\leq 1,$$

where ⌈(*x*) is the gamma function, a generalization of the factorial for non-integer arguments and where ⌈(*n*) = (*n* − 1)! for integer arguments. Setting *β* = 1 gives the standard Mittag-Leffler function E_*α*_(*t*) = E_*α*,1_(*t*) of (22). Another special case is E_1_(*t*) which is the exponential function. In this article, it is in particular the asymptotes of the responses which are important.

### Approximation of the response function

According to [5], the response function may be approximated:

(24) $$\phi_{\mathrm{CC}}(t)∼\{\begin{array}{ll} \frac{1}{\tau \lceil (\alpha )}{\left(t/\tau \right)}^{\alpha -1}, & t\;\ll \;\tau \\ \frac{1}{\tau \lceil (-\alpha )}{\left(t/\tau \right)}^{-\alpha -1}, & t\;\gg \;\tau . \end{array}$$

The small time approximation corresponds to the Curie-von Schweidler law of (1) as mentioned in [12]. An example may also be found in [4] where the current in capacitors followed the Curie-von Schweidler law over days. On first sight, this seems inconsistent with the small time approximation above, but in fact it fits well. The argument is that the capacitors were modeled by a constant phase element. The Cole-Cole model approaches such an element for (*ωτ*)^*α*^ ≫ 1 and *ɛ*_∞_ = 0 and then the capacitance is:

(25) $$C\approx \frac{A}{d}\frac{\varepsilon_{0}\varepsilon_{s}}{{\left(\text{j}\omega \tau \right)}^{\alpha }}.$$

An example in [4] is a polypropylene dielectric with *α*′ of (19) between 0.999 and 1, i.e. *α* between 0 and 0.001, where *α* = 0 is an ideal capacitor. The factor (*ωα*)^*α*^ requires a very large argument to be much larger than one for such a small *α*, e.g. *ωτ* needs to be 10^100^ for *α* = 0.01 in order for the factor to reach a value of say ten. Therefore, even for large frequencies, *τ* has to be very much larger than some days. The point is that even when capacitors followed the Curie-von Schweidler law for as long as several days, this indicates that the small argument approximation of (24) was valid.

**Figure 1:**
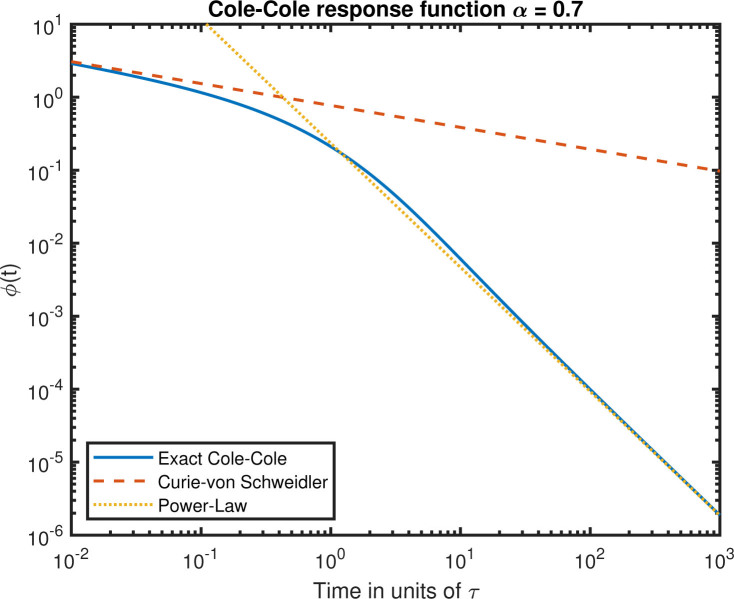
The Cole-Cole response function or current response to a step voltage and its approximations for *α* = 0.7.

The exact expression and the approximations are plotted in Fig. 1 for *α* = 0.7 using numerical code from [13, 14]. The two approximations fit very well for small time and large time respectively.

**Figure 2:**
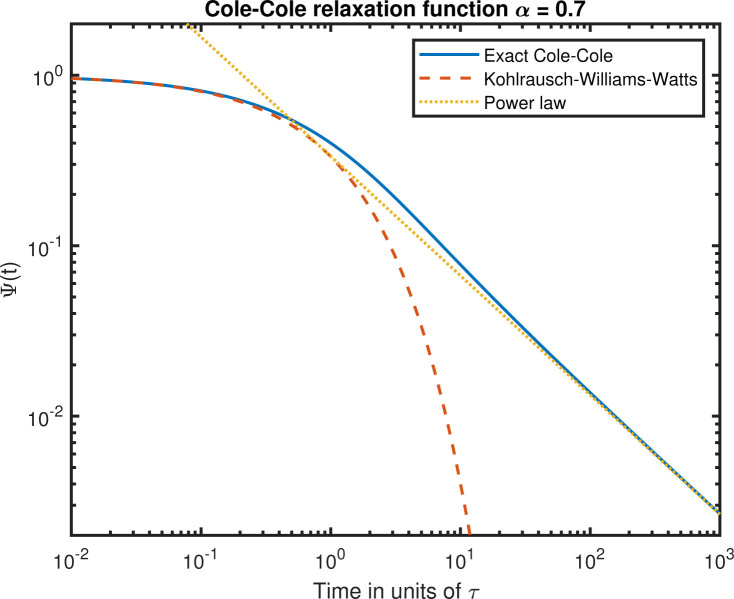
The Cole-Cole relaxation function which is related to the charge response to a step voltage, and its approximations for *α* = 0.7.

### Approximation of the relaxation function

The Mittag-Leffler function with a negative argument raised to a power can also be approximated [8, 5]:

(26) $$\psi_{\mathrm{CC}}(t)∼\{\begin{array}{ll} \text{exp}\left[\frac{-{\left(t/\tau \right)}^{\alpha }}{\left\lceil (\alpha +1\right)}\right], & t\;\ll \;\tau \\ \frac{{\left(t/\tau \right)}^{-\alpha }}{\left\lceil (1-\alpha \right)}, & t\;\gg \;\tau . \end{array}$$

The small time approximation is the stretched exponential or Kohlrausch-Williams-Watt function of (2). For large values of *t* the Mittag-Leffler function approaches a power law [8].The relaxation function along with both its approximations are plotted in Fig. 2. Both approximations fit very well.

### Constitutive law

The Cole-Cole model can also be expressed as a differential equation between D and E. The frequency domain relation building on (19) is:

(27) $$(1+{\left(\text{j}\omega \tau \right)}^{\alpha })D(\omega )=\varepsilon_{0}\varepsilon_{s}E(\omega )+{\left(j\omega \tau \right)}^{\alpha }\varepsilon_{0}\varepsilon_{\infty }E(\omega ).$$

The property of the Fourier transform which was used in transforming from (17) to (18) is that (j*ω*)^*n*^ transforms to the nth order derivative for integer *n*. This property is generalized in fractional calculus to non-integer orders *α*. This demonstrates the close relationship between power-laws and fractional calculus [11]. This results in this constitutive equation for the Cole-Cole model:

(28) $$D(t)+\tau^{\alpha }\frac{{\text{d}}^{\alpha }D(t)}{\text{d}t^{\alpha }}=\varepsilon_{0}\varepsilon_{s}E(t)+\tau^{\alpha }\varepsilon_{0}\varepsilon_{\infty }\frac{{\text{d}}^{\alpha }E(t)}{\text{d}t^{\alpha }}.$$

This is the electrical equivalent of the fractional Zener model in linear viscoelasticity [7]. Fractional calculus has become an important research area in recent decades both in mathematics and in physics, despite its roots long ago [15]. One feature is that non-integer derivatives have memory. The details are beyond the scope of this paper, but hopefully its role in describing the Cole-Cole model may be a motivation for delving into it in e.g. the two books just cited.

### Beyond the Cole-Cole model

There are several alternatives to the Cole-Cole model such as the Cole-Davidson and Havriliak-Negami models. The latter is the more general one:

(29) $$\tilde{\varepsilon }(\omega )=\varepsilon_{\infty }+\frac{\varepsilon_{s}-\varepsilon_{\infty }}{{\left(1+{\left(\text{j}\omega \tau \right)}^{\alpha }\right)}^{\beta }},\;\;\;0\;<\;\alpha \;\leq 1,\;\;\;0\;<\;\beta \;<\;1,$$

where *β* = 1 gives the Cole-Cole model and *α* = 1 gives the Cole-Davidson model. All three models yield an ideal capacitor in parallel with the constant phase element of (25) in the limit of a large *τ*. These models are also analyzed in [5] and the main thing to note is that the Curie-von Schweidler law and the Kohlrausch-Williams-Watt function fit the asymptotes of these models just as well as they fit the Cole-Cole model [12]. The link between the constant phase element, these early empirical results, and the Cole Cole model is therefore not unique.

## Conclusion

The familiar frequency domain expression for the Cole-Cole model of order *α* can also be expressed in the time domain. The response function, which is related to the current response to a voltage step excitation, is expressed with a two-parameters Mittag-Leffler function. Its asymptote for small time is a power-law function which corresponds to the Curie-von Schweidler law. The relaxation function, which describes the charge response, is given by a one-parameter Mittag-Leffler function where the asymptote for small time is the stretched exponential or Kohlrausch-Williams-Watt function. For the Debye model, both these responses are given by exponential functions in time. The Debye model is also equivalent to a first-order differential equation between electric field, E, and the displacement field, D. This generalizes to a fractional differential equation with non-integer derivatives of order *α* for the Cole-Cole model.
